# 3D Dataset of binary images: A collection of synthetically created digital rock images of complex media

**DOI:** 10.1016/j.dib.2022.107797

**Published:** 2022-01-06

**Authors:** Javier E. Santos, Michael J. Pyrcz, Maša Prodanović

**Affiliations:** Hildebrand Department of Petroleum and Geosystems Engineering, The University of Texas at Austin, Austin, TX, USA

**Keywords:** Sphere-packs, Fractures media, Catalyst layers, Shales, Vuggs

## Abstract

Digital rock images are computational representations that capture the geometrical complexity of systems present ubiquitously in nature. In recent years, their use has become widespread due to the increasing availability of repositories, and open-source physics simulators and analysis tools. Here, we present a dataset of 3D binary geometries in a standardized format that represent a wide variety of geological and engineering systems. Our data is freely available at [1].

## Specifications Table

**Table:** 

Subject	Computers in Earth Sciences
Specific subject area	3D Digital Rock Images
Type of data	Image
How data were acquired	Computer simulations
Data format	3D arrays in HDF5 format
Parameters for data collection	After carrying-out the simulation to generate each image, all the isolated pore bodies were removed and a check was performed to assess connectivity along the third coordinate axis
Description of data collection	The images are presented as binary 3D arrays of zeros and ones. The former represent the pore-space and the later the solid phase.
Data source location	Institution: The University of Texas at Austin
Data accessibility	Repository name: Digital Rocks Portal (DPR) Data identification number: 374 Direct URL to data: https://www.digitalrocksportal.org/projects/374 The code to create this dataset is part of the MPLBM-UT library [2].

## Value of the Data

• The dataset includes 75 3D geometries (rock samples) representing a wide variety of depositional systems and diagenetical processes. This data has been used in a number of research articles [3], [4], [5], [6], [7], [8], [9], [10], [11].
• The collection is standardized with a high-performance file format (HDF5) that integrates its metadata (for portability), making it accessible with any programming language and popular scientific-visualization software. All the samples were post-processed to ensure 6-connectivity which allows flow simulations to converge faster.
• Researchers and users involved in direct simulations methods of physical processes can benefit from these images for benchmarking their simulations and inferring new physical insights with a standardized dataset. AI researchers will utilize this dataset of complex geometries to test supervised and unsupervised classification algorithms.
• The images can be used as inputs for physics-based simulations (e.g., single- and multi-phase flow [2], [12], [13], particle transport, nanoconfinement, electrical conductivity, and solid mechanics) to get new physical insights (e.g., scaling relationships, surrogate modeling, etc.), geometrical analysis (e.g., geostatistics, spatial statistics, etc.) [14], train supervised/unsupervised deep learning models, on samples that are representative of different subsurface settings.

## 1 Data Description

Flow and transport in porous media are critical to understand the geological processes of rock formation and applications such as the management of groundwater resources, carbon sequestration, enhanced oil recovery, and contaminant transport. Typical geological systems are composed of a broad spectrum of porous media with features such as porosity and permeability varying by orders of magnitude within an individual system. An example application is to utilize a digital rock as an analog to derive the relationship between porosity and permeability to impute missing permeability data from porosity data points, which are widely available.

The term ‘digital rock’ refers to a computational model of a porous medium in 2D or 3D whose source is either an actual image of a rock or soil (e.g., an x-ray microtomography image), or it is based on a model (e.g., calculated via process-based simulations of sediment transportation and deposition stochastic realization from a geostatistical model). These are commonly formatted in a regular grid or mesh that allow their use in a numerical simulator to forecast a transport process or property of interest. These simulations are then post-processed to obtain a bulk property that characterizes the rock in question, and this research area is often referred to as digital rock physics, or digital core analysis. Digital Rocks Portal [15] provides a curated library of digital rocks representing a wide variety of domains ranging from imaged biofilms in soil to texturally equilibrated models of salt pore spaces. Digital Rocks Portal stores both actual images from a variety of imaging techniques and representative porous media models. Digital Rocks Portal is organized in units called *projects*.

The dataset collection (Digital Rocks Portal project) described here provides 3D porous and fractured models that systematically vary a range of their properties and can be used for benchmarking simulation algorithms, including systematic training of deep learning algorithms predicting transport properties [10], [11]. Datasets are in the form of binary images that have designated pore (void) and solid (grain) numerical cells or voxels, and are organized in 10 groups (or *samples*, in Digital Rocks Portal jargon). Many have already been used in previously published work though not necessarily shared. The groups are described as follows:

**Group 1** contains discretized representation of experimental measurement of a packing of identical spheres (Sample 1, the original is stored at the portal [16]). This sample is then systematically changed to mimic diagenesis processes. One morphological sphere dilation and six morphological erosions are numerically performed to obtain samples with higher (Sample 0) and lower porosity (Sample 2–8) respectively, but that still exhibit a similar pore structure to the original sample. These samples come in both 256${}^{3}$ and 480${}^{3}$.

**Group 2** contains self-similar fractures running through the middle of the volume. This group contains five fractures with varying surface roughness representing different materials. These five fractures are downscaled three times to create fractures with smaller aperture that still exhibit the same degree of surface roughness. All the downscaled fractures have the same mean aperture (hence, porosity) respectively.

**Group 3** contains packings of randomly placed spheres. Unlike Group 1, these spheres are allowed to overlap slightly to achieve a target porosity. These images are well-suited to represent catalyst layers [17]. This group contains samples with six porosities (10, 15, 20, 25, 30, and 35%) with three realizations each (since the algorithm uses a random number seed to start the simulation). **Group 7** contains samples created with the same algorithm and with different seeds.

**Group 4** contains two process-based reconstructions of nanoporous organic matter in shale [5]. These samples have a porosity of 20 and 30%.

**Group 5** contains four fractured samples. Unlike Group 2, the fractures have an adjacent permeable matrix [3], [4].

**Group 6** contains two samples with a linear gradient in the porosity of their slices in the z-direction.

**Group 8** contains a realistic single fracture on a solid domain. The roughness of this sample is heterogeneous and the aperture field is not self-affine.

**Group 9** Randomly placed overlapping spheres with an embedded synthetic fracture along the z-axis.

**Group 10** Voxelized sphere packs where a certain number of solid grains were removed to simulate a vuggy domain.

**Fig. 1:**
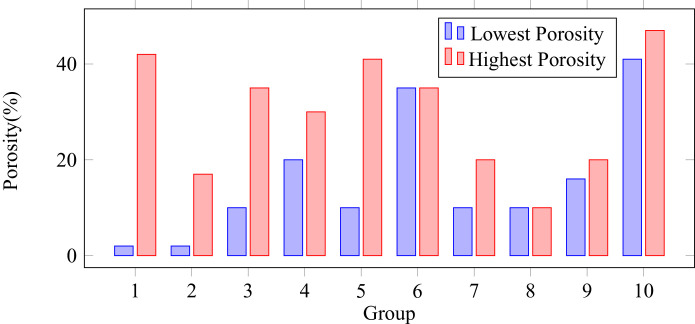
Lowest and highest porosity sample values in each group.

**Fig. 2:**
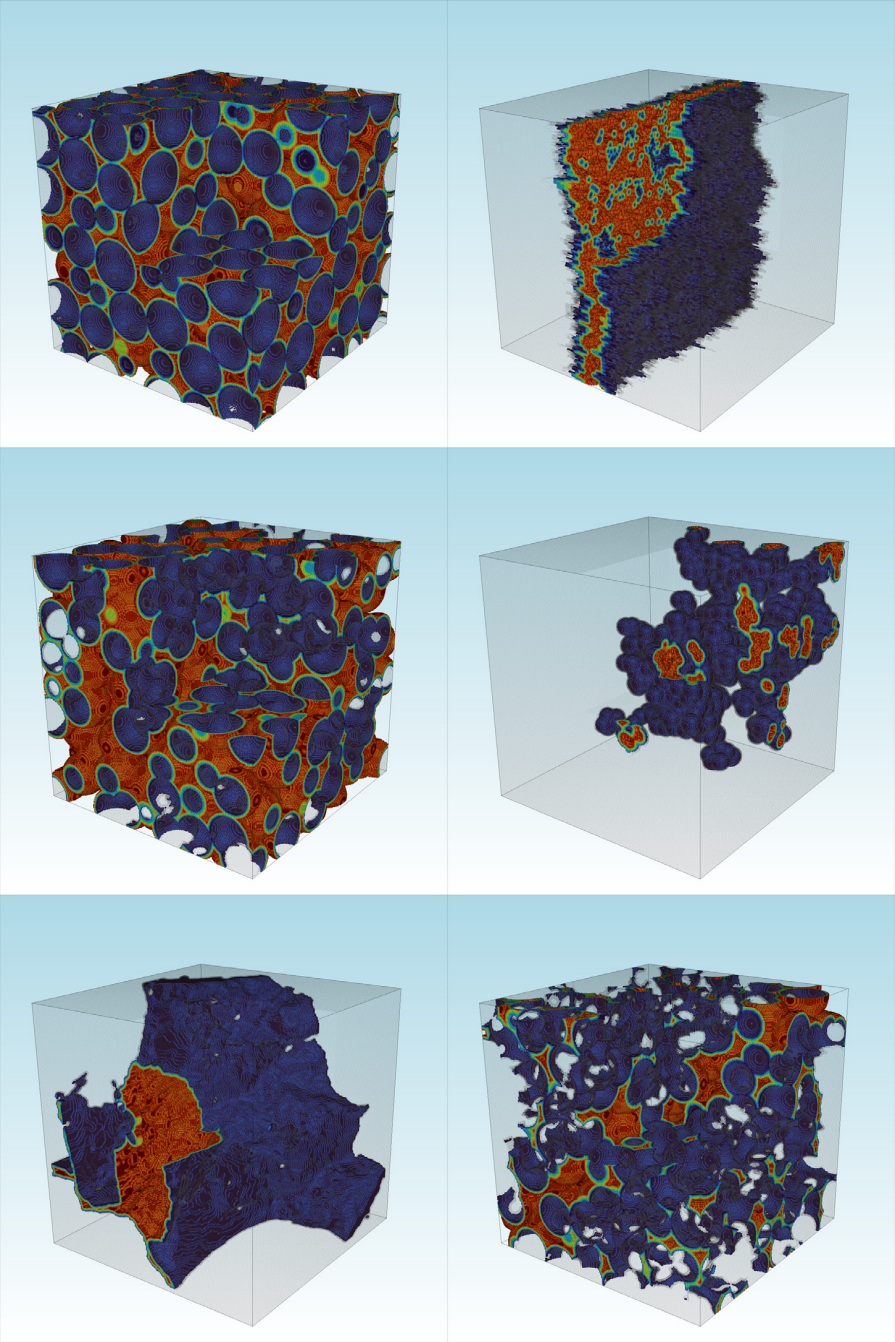
3D plots of dataset samples. Each subplot shows the Euclidean distance of the pore space for visualization purposes. The code to reproduce this figure is shown in Listing 1.

The highest and lowest porosity values (defined as number of zero-entries over image size) in each group can be visualized in Fig. 1. A selection of samples is shown in Fig. 2.

**Listing 1:**
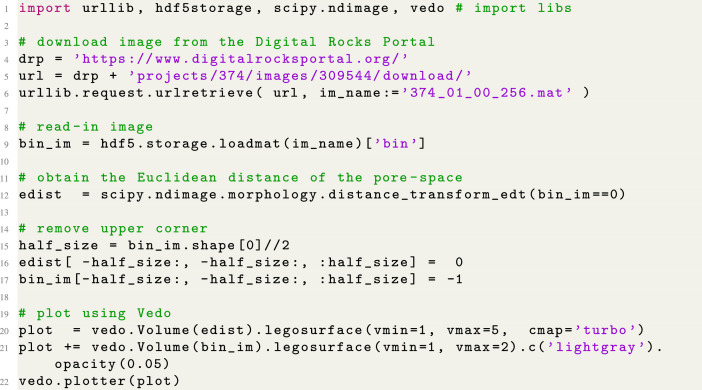
Python code to download and plot the binary images as shown in Fig. 2.

## 2 Experimental Design, Materials and Methods

All the image groups were created synthetically to represent different natural settings. The main motivation to create this dataset was to have a diversity of benchmark samples to perform transport simulations and geometric analysis. All the samples are discretised as 256${}^{3}$ volumes and some have an additional 480${}^{3}$ volume. All of these are presumed unit-less and can be scaled to any physical length of interest (for instance, spheres diameters can be scaled by a relevant mean grain diameater). The pore-space is represented with zeros and the solid matrix with ones. Most of the images have been used in projects related to a wide range of earth-science applications.

## Declaration of Competing Interest

The authors declare that they have no known competing financial interests or personal relationships which have, or could be perceived to have, influenced the work reported in this article.
